# Fragments of Medical Science and Art, an Address by Henry J. Bigelow, M. D., of Boston

**Published:** 1846-05-01

**Authors:** 


*Fragments of medical Science and Art. An address by Henry J. Bigelow, M. D. Boston, Mass.*—This is the production of a mind above the common order. It evinces learning, philosophical meditation, and vigorous thought, as well as cultivated habits of composition. The views of the writer especially on the position of Hypotheses in philosophical investigations, are, in our opinion, just, and practically important. We have marked some passages for quotation hereafter.

